# Cultivation of Positive Psychological Quality of College Students' English Learning Under the Online and Offline Teaching Mode During the Epidemic

**DOI:** 10.3389/fpubh.2022.929027

**Published:** 2022-08-17

**Authors:** Han Yu, Xinguo Li

**Affiliations:** School of Foreign Languages, Anhui Polytechnic University, Wuhu, China

**Keywords:** post-pandemic era, positive psychology, BP neural network, health education, electronic games

## Abstract

During the COVID-19 pandemic, long-term isolation and loneliness will cause college students' psychological fluctuations. Especially in online teaching, the lack of communication for a long time has led to a greatly reduced learning enthusiasm of college students. Therefore, this paper aims to explore the cultivation methods of the positive psychological quality of college students under the epidemic situation through the research on the positive psychology of college students' English learning. Aiming at the psychological status of college students, this paper focuses on analyzing the relationship between social support, psychological capital, and psychological health to explore more targeted ways of cultivating positive psychology. Because of the online and offline teaching mode, this paper focuses on analyzing the support environment of the online teaching mode, and analyses the current forms of English teaching. Experiments show that the direct path from psychological capital to mental health is not significant. However, the mediating path of psychological capital to mental health through social support was significant (*p* < 0.001). It shows that social support plays a complete mediating role, and the effect size of the mediation model reaches 49.70%. It shows that the current college students' English learning positive psychological quality is not high. In response to this, it is necessary to strengthen the tendency and ability to use social support and use the family environment to communicate more to achieve the cultivation of positive psychological quality.

## Introduction

At the end of 2019, the outbreak of the novel coronavirus pneumonia caused by the novel coronavirus broke out across the country. On January 30, 2020, the World Health Organization declared the novel coronavirus pneumonia outbreak a “public health emergency of international concern.” More than 30 provinces, cities and regions have launched first-level responses to major public health emergencies. The Ministry of Education has decided to postpone the start of the spring semester of 2020, and carry out online teaching of “suspending classes without stopping.” The COVID-19 pandemic has ushered us into a brave new world of business and management. The nature, values, processes, systems and more of our workplace are changing dramatically at a rate we could not have imagined before. New work patterns, occupations, and even industry boundaries have emerged in a relatively short period in the form of a global movement. Organization members' attention shifts from gain, competition, efficiency, and scale to a fundamental desire for survival, resilience, and regeneration. Although it can look forward to the gradual arrival of dawn and the cessation of the pan-human social catastrophe, the transformation seems unstoppable. New technologies, such as artificial intelligence, blockchain, VR/AR, Internet of Things, big data analysis, etc., lead the workplace to reengineering. People went from being interested in this transformation to being familiar with it, and moving forward firmly.

The epidemic has caused great damage to China's economy and people's lives in a very short period. And in the early stage of the prevention and control of the new crown pneumonia epidemic, that is, the incubation period and the outbreak period of public opinion, the negative public opinion is clamoring, bringing an additional burden to the prevention and control of the epidemic. In order to effectively block the threat posed by the epidemic to schools and teachers, and students, colleges and universities are encouraged to change their teaching thinking, promote the reform of learning methods, and complete the migration and integration of offline teaching to online teaching. However, such isolation will undoubtedly bring a greater psychological burden to college students. Therefore, it is particularly important to cultivate the positive psychological quality of college students' English learning.

It explores the current situation and problems of “Internet + Education” online teaching. The optimization is an inevitable requirement of curriculum teaching reform and innovation. This research aims to analyze and study the positive psychological quality, core elements, teaching mode and predicament of online teaching of English learning for college students under the background of COVID-19 and propose development strategies. Specifically: (1) Scientifically determine the connotation of “Internet + education,” and explore the value of “Internet + education” from the perspective of theory and practice. (2) The core elements of “Internet + Education” are analyzed; the typical models and characteristics of large-scale “Internet + Education” in the context of COVID-19 are summarized; the “Internet + Education” model is constructed, and its application principles are pointed out. (3) because of the current situation and dilemma of college students' psychological pressure under COVID-19, the development strategy of college students' positive psychological cultivation is proposed.

## Related Work

The positive psychology of college students is the basis of their normal life and study, so there are many analyses of the positive psychology of college students' study. Katz J. studied the relationship between positive psychology and memory, etc. He found that self-regulation training was beneficial in improving memory, concentration, and executive function and reducing levels of anxiety, stress, and depression (1). Shuang L. believed that with the diversification and richness of video game platforms, video games have an increasing impact on people's mental health and behavior, thus becoming a hotspot in psychological research (2). Farid C. believed that although positive psychology interventions (PPIs) have shown beneficial effects on the mental health of non-clinical populations, the current literature is inconclusive about their effectiveness in clinical settings. He aimed to examine the effects of PPIs on wellbeing (primary outcome), depression, anxiety and stress (secondary outcome) in clinical samples with psychiatric or medical illnesses (3). Chodkiewicz A. R. believed that the epidemic trend is accompanied by the rise of positive psychology, which is changing the concept of youth, education and development (4).

However, it can be found that the current research on the psychology of college students is more aimed at purely psychological aspects without taking into account the background of the current epidemic. The purpose of Goldman D. T. was to assess the current evidence on the use of telemedicine in the surgical subspecialty during the COVID-19 pandemic. He argued that telemedicine has increased access to care for surgical patients during the COVID-19 pandemic, but whether this practice will continue post-pandemic remains unknown (5). The purpose of Szturo M. was to identify the threat of default risk of commodity-related companies in European equity markets. He determined that the default risk of companies listed on multiple stock exchanges follows the Merton model by comparing bankruptcy probabilities for the time intervals from January 1, 2019 to June 30, 2019 and from January 1, 2020 to June 30, 2020. Calculations are based on data from the Wall Street Journal database (6). The purpose of Kiran D. was to study knowledge, attitudes and practices of physical distancing in the general population and to identify gaps in knowledge and practice of physical distancing among them. He employed a cross-sectional study design using the snowball sampling technique. His research helped to identify knowledge-practice gaps (22.2%) and behavioral patterns that would further aid in implementing effective interventions (7). It can be found that although the relevant research has made a particularly comprehensive and in-depth analysis of the psychological characteristics of college students, there is still a lack of research on the positive psychological quality of college students, especially the characteristics of the current epidemic.

## Psychological Quality of College Students Under the Epidemic

### Online and Offline Teaching Mode and English Learning During the Epidemic

During the epidemic prevention and control period, “Internet + education” has become the main solution for “suspending classes without stopping learning.” Online teaching has become the most widely used form of “Internet + education” in the context of COVID-19. The implementation of large-scale online teaching involves multiple elements. All elements cooperate with each other to support the smooth development of online teaching.

**Figure 1:**
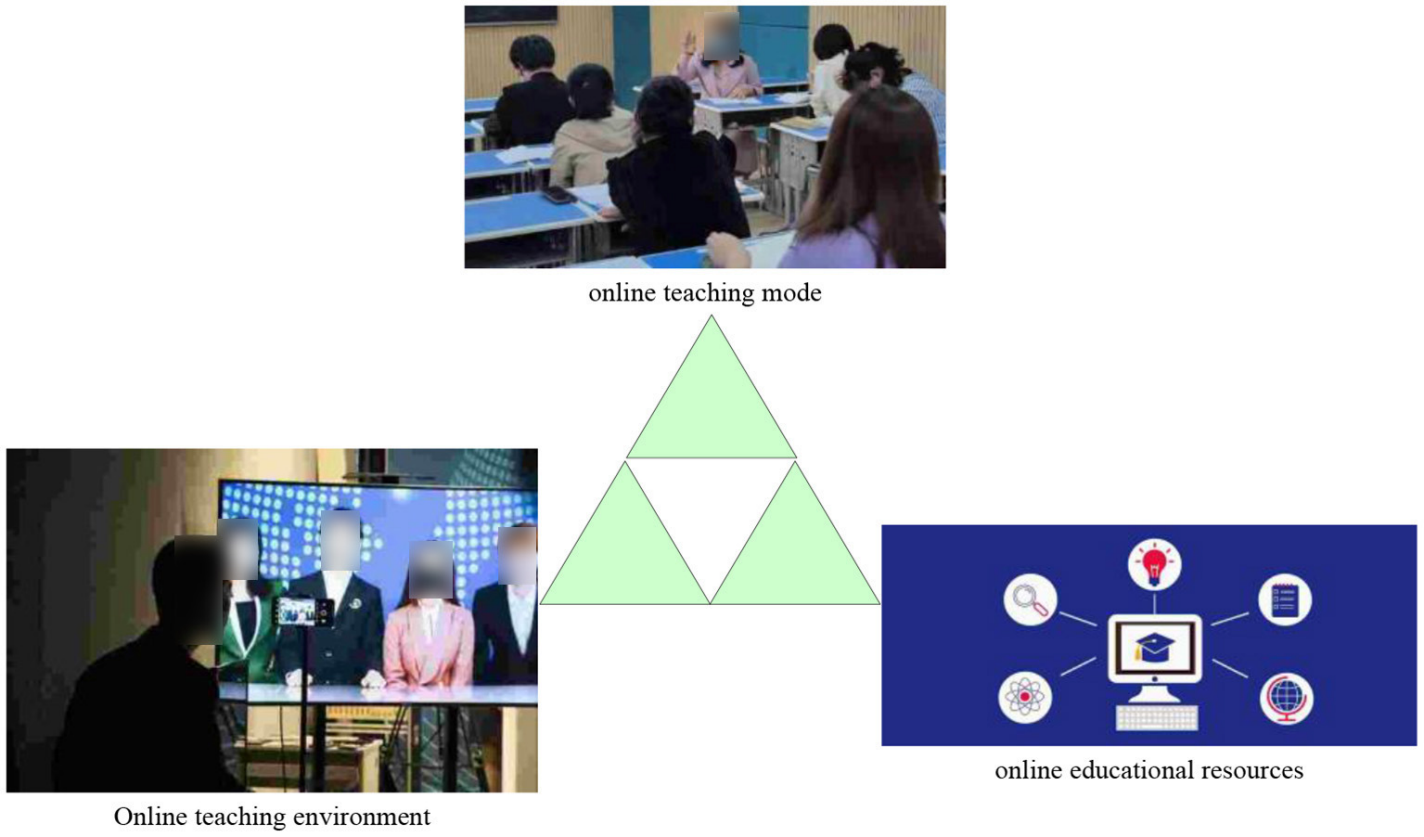
Core elements of “Internet + education”.

“Internet + education” in the context of COVID-19 can be regarded as the deployment and operation of a macro system involving many elements. For example, education administrative departments and schools in the category of teaching management system; enterprises, teaching platforms, teaching resources, learning tools, etc. in the category of a teaching support system (8); teachers, parents, and students in the category of teaching implementation. By analyzing and summarizing its constituent elements in the existing literature and cases, it is concluded that the three core elements of “Internet + education” in the context of COVID-19 are: “basic environment, teaching support, and teaching mode” (9, 10), as shown in Figure 1.

As a bridge connecting teachers and students and carrying various teaching resources, the teaching platform is very important for developing online teaching activities. Under the epidemic situation, online teaching platforms are in a state of blooming. By investigating various live broadcast platforms during the epidemic, this paper summarizes two main types of online teaching platforms: live-streaming online teaching platforms and resource-based online teaching platforms.

**Figure 2:**
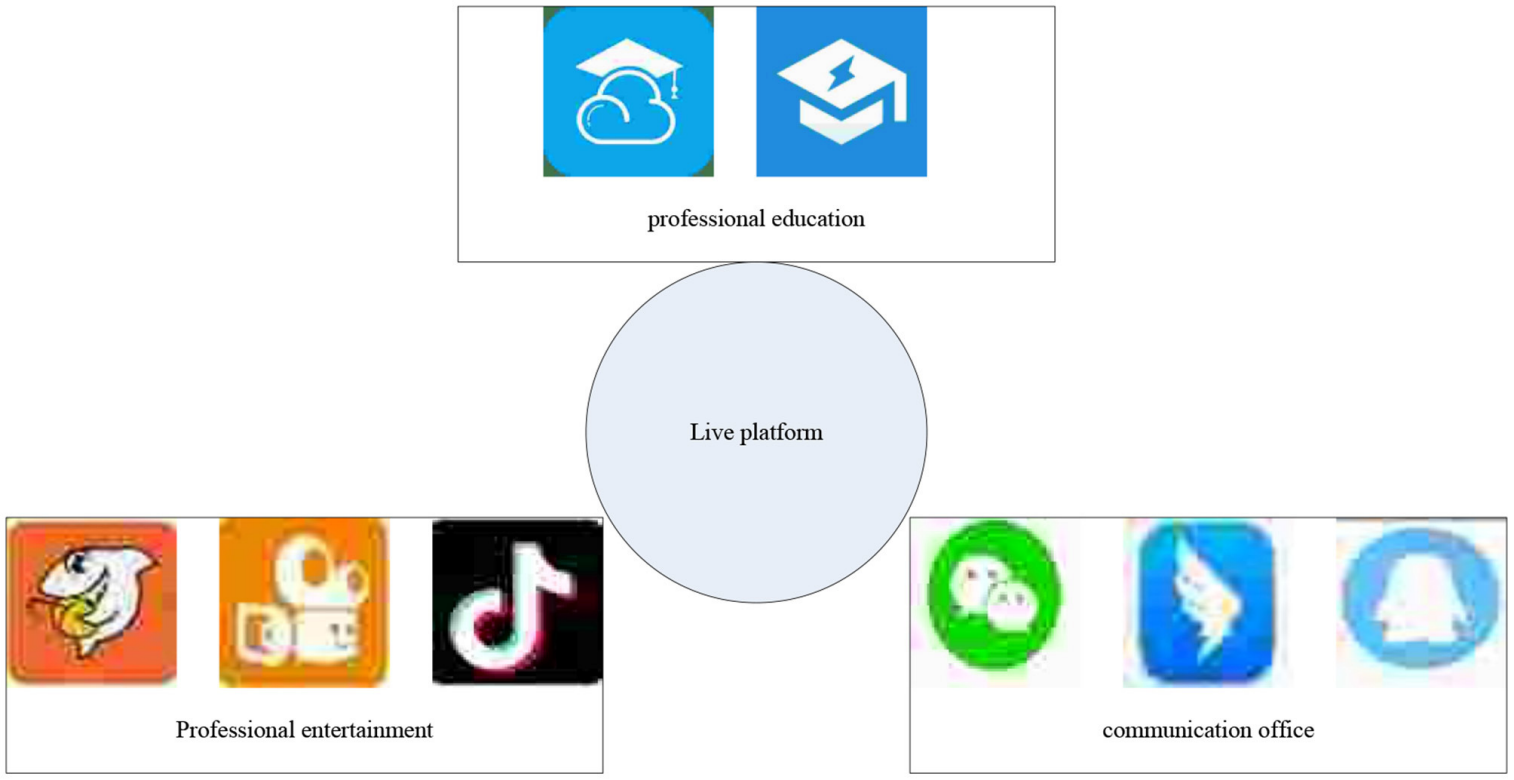
Common live online teaching platforms.

The characteristics of live broadcast platforms are that the teaching content is broadcast in real-time, and students and teachers can achieve online synchronous classrooms. The live online teaching platform is aimed at large classes and can give full play to allocating and sharing resources. Real-time interaction in teachers' teaching is beneficial to increasing students' participation in learning activities. The live teaching platform can more easily restore the offline classroom form online, which is conducive to students' mastery of learning content and methods (11, 12). Common live online teaching platforms are shown in Figure 2.

**Figure 3:**
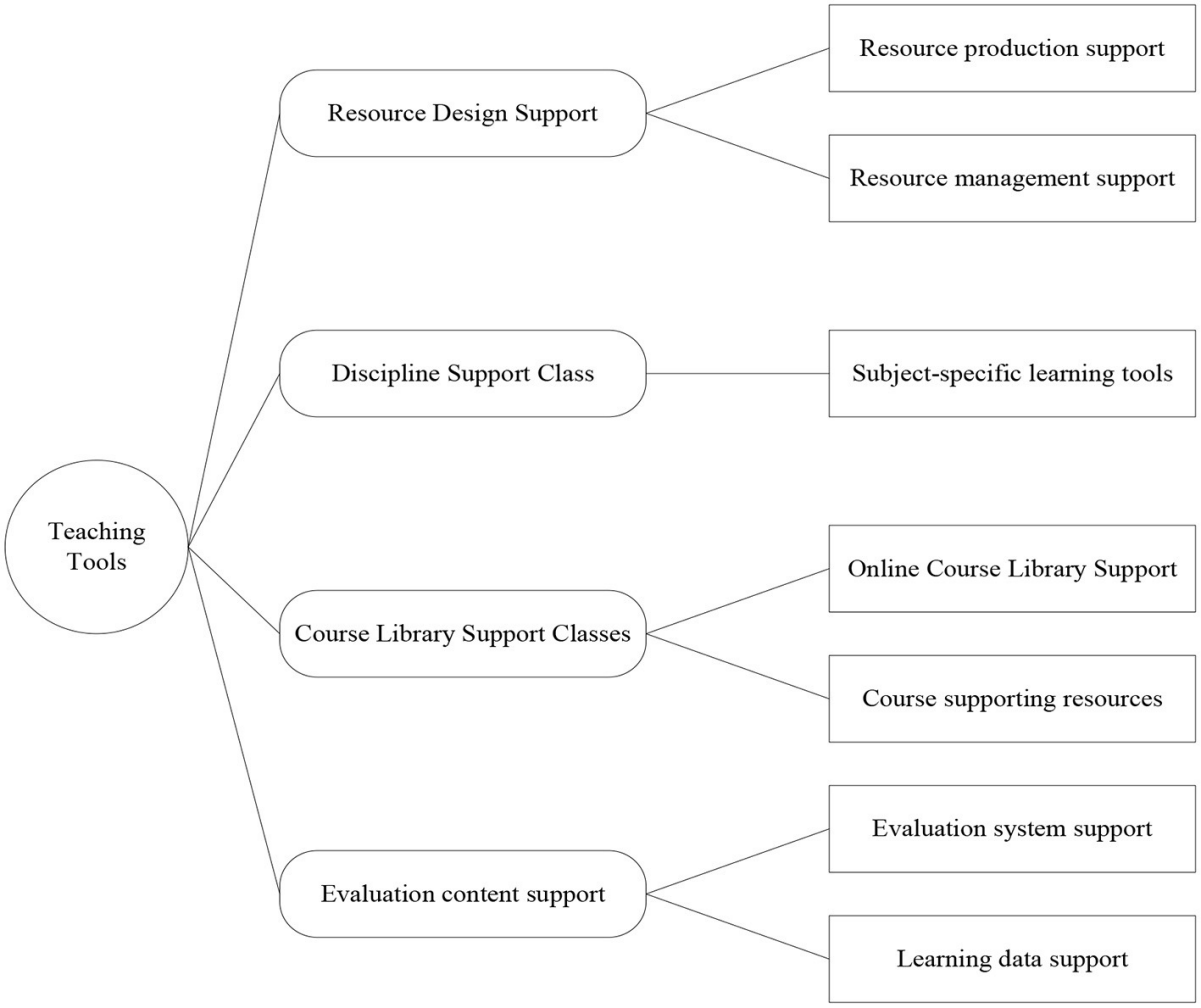
Categories of online teaching tools.

The online teaching software is classified based on analyzing and summarizing various online teaching tools. According to various online teaching software characteristics, it can be divided into resource design support tools, subject support tools, course library support tools, and evaluation content support tools. The specific classification of online teaching tools is shown in Figure 3.

**Figure 4:**
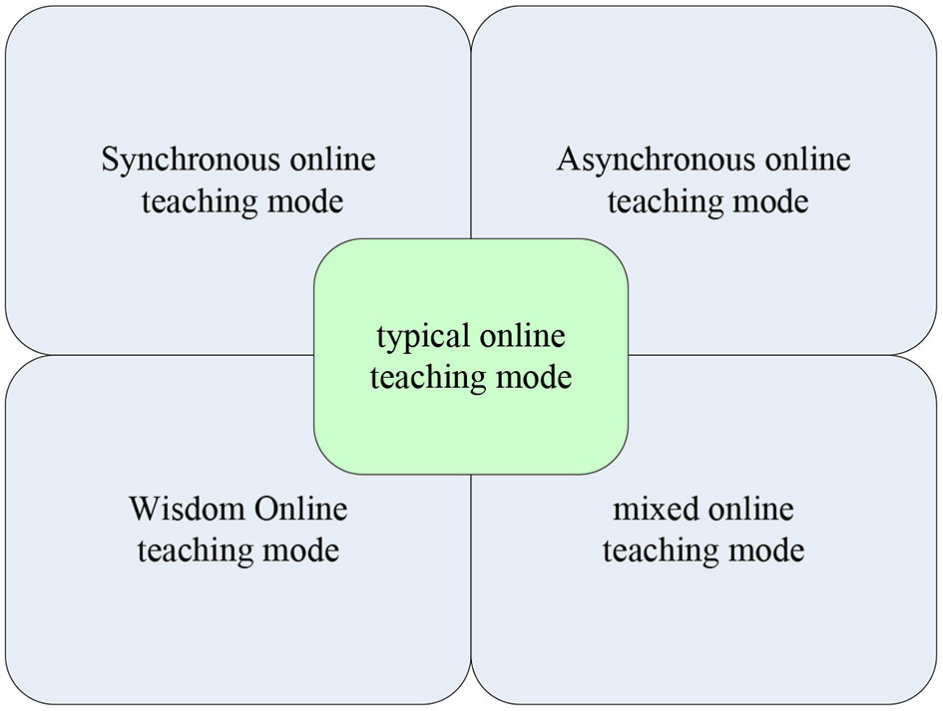
Typical online teaching mode under the epidemic.

The typical online teaching modes shown in Figure 4 are: synchronous online teaching mode, asynchronous online teaching mode, smart online teaching mode, and hybrid online teaching mode.

The online teaching of “Internet + education” is different from traditional classroom teaching and has its own characteristics. This study considers online teaching as a new teaching method based on the information technology environment. The information technology environment is a distinctive feature of online teaching. Teachers and students separated by time and space need various learning supports to rebuild the learning community environment in online teaching. Furthermore, large-scale and long-term online teaching differs from traditional classroom teaching (13, 14).

### Psychological Characteristics of College Students Under the Epidemic

**Figure 5:**
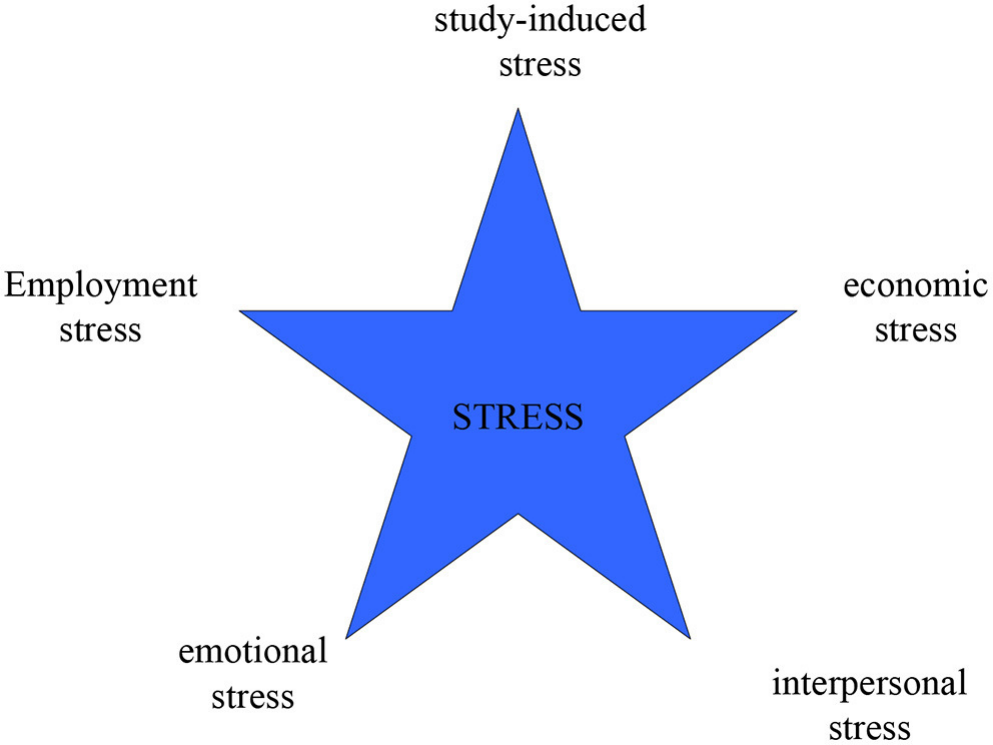
Sources of psychological stress of college students.

Understanding anxiety requires dialectical thinking. Anxiety is a personal experience of unpleasant emotions, but if it develops into a serious psychological state, it can cause obstacles to personal action. Appropriate anxiety can make students work hard, but excessive anxiety can make college students depressed or even seriously affect their adaptability to society. Their mental health is directly related to the quality of school personnel training and the university's long-term development. They are not only the happiness and hope of the family but also the development and progress of the society. The research field of this paper will aim to expand the applicability of anxiety theory and thus will also help to enrich mental health education in colleges and universities. In the era of the epidemic, the sources of psychological stress of college students are shown in Figure 5.

Against the background of unresolved unemployment stock, domestic college graduates are still increasing every year. According to data released by the Ministry of Education, in 2020, graduates from ordinary colleges and universities nationwide reached 8.74 million. However, as the sudden new crown epidemic has caused a greater impact on the social and economic downturn, the employment difficulty coefficient of college graduates has increased significantly, and the employment situation has become more severe. During this special period, many offline presentations and recruitment have been suspended due to the epidemic, and many recruitment activities have been changed to online. Circumstances like this have had a huge impact on the way communication is done in employment. For example, traditional offline recruitment has been changed to online interviews, and communication methods have become more virtual. This is a new challenge for the employment of University graduates. According to the employment situation data of fresh college graduates released by Liepin Big Data Research Institute, <30% of graduates have signed contracts for employment.

**Figure 6:**
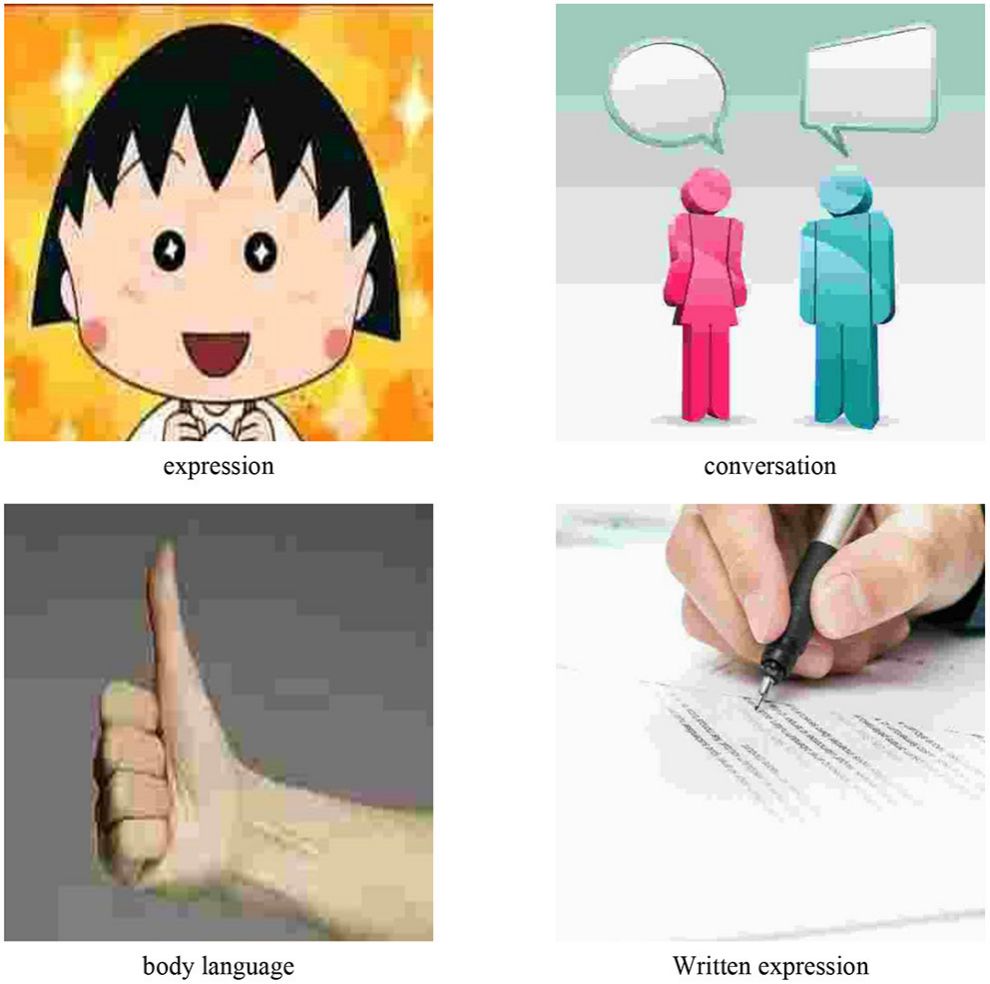
Types of communication.

Communication from different angles has different definitions, and communication in practical applications also has different modes. As the name suggests, formal communication is used in more formal situations. In other informal cases, it is informal communication. From the perspective of the carrier of information, communication can be divided into verbal communication and non-verbal communication. Verbal communication mainly refers to oral or written communication, including oral and written communication. Non-verbal communication requires the help of certain media to achieve the purpose of information transmission, including expressions, body behavior and other forms (15, 16). From the perspective of communication feedback results, communication can be divided into one-way and two-way. One-way communication refers to the transmission of information in one direction, and two-way communication belongs to the two-way transmission of information. The details are shown in Figure 6.

**Figure 7:**
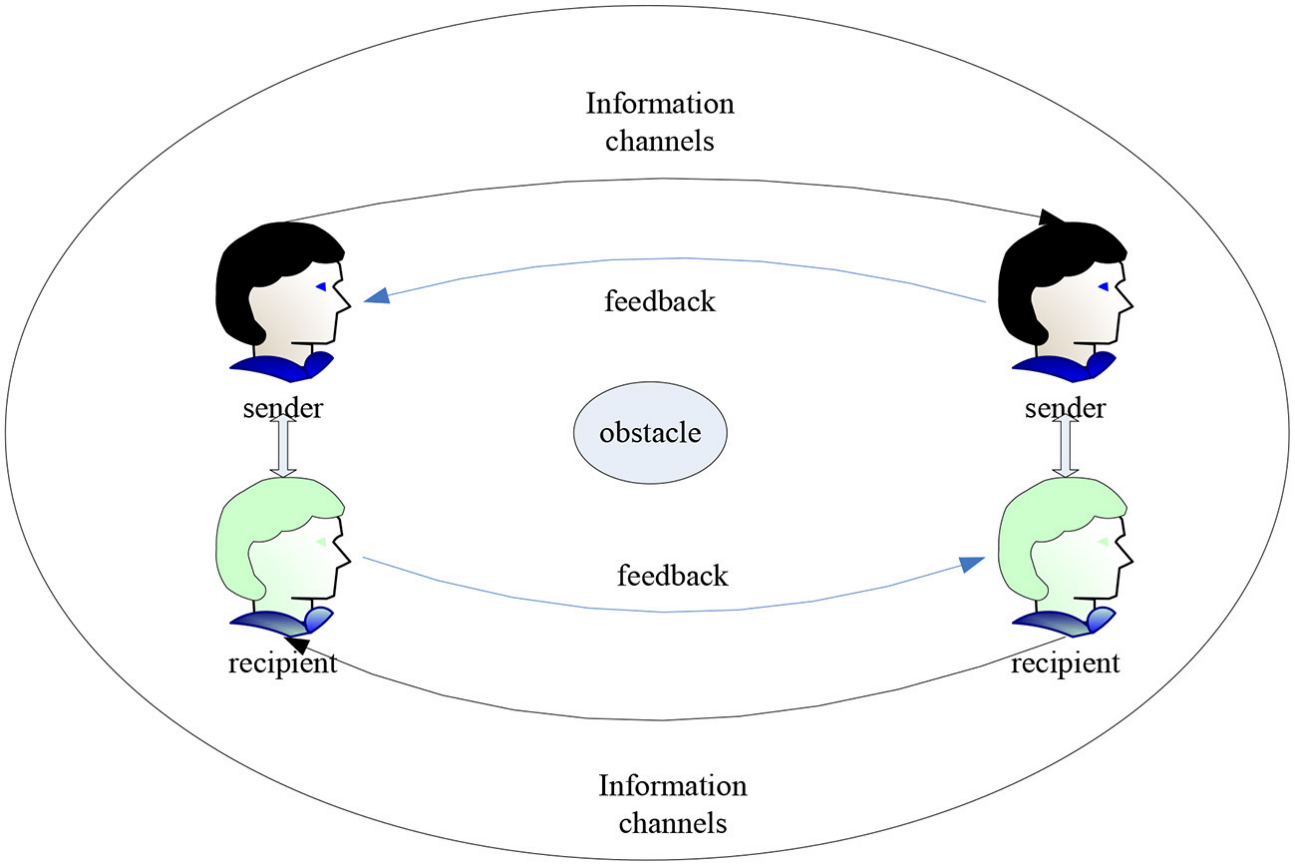
Elements of interpersonal communication.

Figure 7 shows the communication interaction model in which information is transmitted in a certain situation, and the meaning of information transmitted in different situations may be different. Different communication channels convey different information. Nowadays, communication channels are becoming more and more diverse, such as on-site communication, voice calls, WeChat and other forms. Generally speaking, in the process of face-to-face communication, the received information can be analyzed from multiple angles, which is also a common method. In addition, according to the different subjects of communication, communication is divided into self-communication (internal communication), interpersonal communication (communication with others), group communication (communication with multiple people), corporate communication and other forms. The environment for college graduates is relatively simple. The main forms of communication are self-communication, interpersonal communication with others and communication between groups. It's just that the barriers to communication are different in different communication situations. Due to the development of the Internet and the epidemic, the job search of college graduates has changed from the traditional face-to-face method to diversified (17).

**Figure 8:**
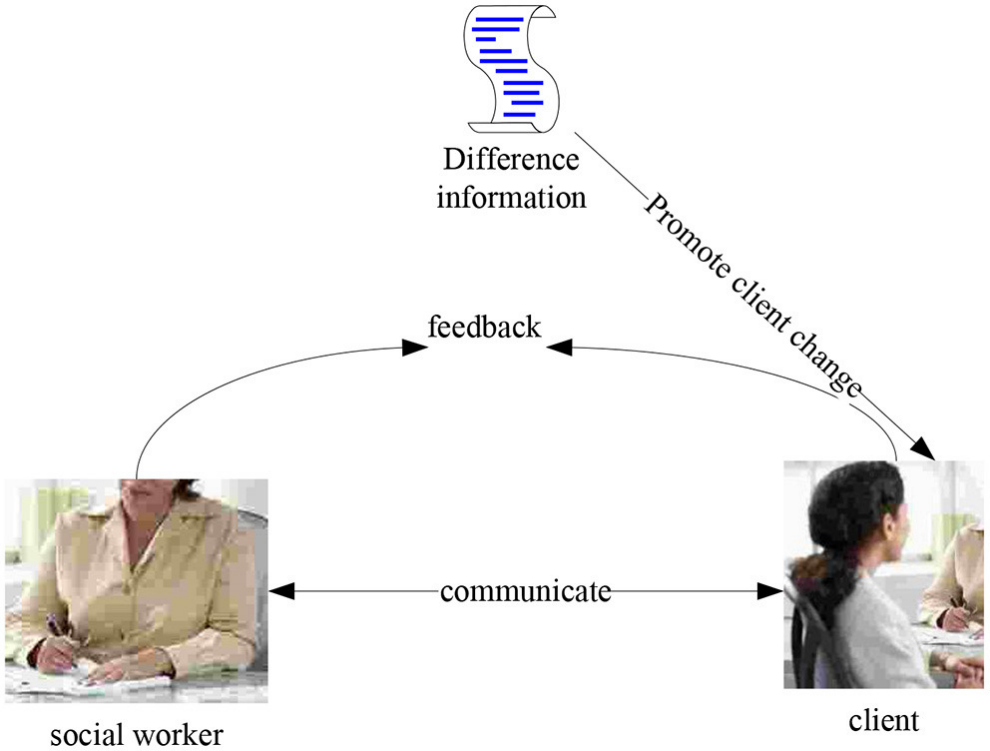
Communication intervention model.

As can be seen from Figure 7, communication theory is widely used in management and psychology. In contrast, in social work, communication theory has always focused on the study of the individual's psychological state while ignoring individual behaviors in different situations, such as individual communication patterns. The core point of communication theory is to understand the interaction between the client and others in a certain situation and continuously explore and cultivate the client's good communication style. Therefore, this theory focuses not just on the individual but also on the interactions around the individual. It is also an individual's attitude and behavioral choices in the face of the network's social structure. In social work, communication theory is used as the theoretical basis for a service intervention. Continuously asking for different information in a loop helps service objects establish correct interpersonal communication methods. The specific intervention communication process is shown in Figure 8. It can be seen that this process includes a large number of information selection and flow, coordination mechanism between actors, repeated construction of consensus power, individual plans and their self-adjustment (18).

### SVM Classification Algorithm

In stress relief, it is often necessary to determine the source of the stress to make a better-targeted analysis. Semi-supervised support vector machine (S3VM) is a typical semi-supervised machine learning algorithm. Support Vector Machine (SVM) is a classification algorithm. The classification test is determined using the criteria of minimum empirical risk and maximum “marginal.” In semi-supervised learning, there are labeled datasets:

(1) $$\begin{array}{r} \left\{\left(x_{i},y_{i}\right)|1\leq i\leq L\;\right\},x\in R^{n} \end{array}$$

Unlabeled dataset:

(2) $$\begin{array}{r} \left\{x_{i}|L+1\leq i\leq L+U\;\right\} \end{array}$$

The training set can be viewed as the union of the labeled and unlabeled datasets. Semi-supervised Support Vector Machines (S3VM) have the same decision function as Support Vector Machines (SVMs):

(3) $$\begin{array}{r} f_{\theta }\left(x\right)=\omega \cdot \Phi \left(x\right)+b \end{array}$$

Among them:

(4) $$\begin{array}{r} \theta =\left(\omega ,b\right) \end{array}$$

It is all the parameters of the model, and b is the bias, which is the feature mapping function, which needs to be specifically designed according to different tasks. Semi-supervised Support Vector Machines (S3VM) fit the clustering assumption. The clustering hypothesis states that data samples in the same cluster are likely to have the same label. This assumption is also the key to many other successful semi-supervised learning algorithms. The clustering hypothesis states that the classifier should try to keep the classification boundary (decision boundary) away from areas with high data density. The optimization goal of the Semi-Supervised Support Vector Machine (S3VM) is to maximize the margin from the sample to the classification interface of the classifier (19, 20). The main idea of this idea is to find a support vector machine (SVM) classifier under a certain conditional constraint, and the constraint condition can make the margin from the unlabeled sample to the classification boundary (decision boundary) as large as possible. According to the above ideas, the optimization objective of a semi-supervised support vector machine (S3VM) is as follows

(5) $$\begin{array}{r} {\text{min}}_{w,b}\frac{1}{2}∥\omega^{2}∥+C\sum_{i=1}^{L}\xi_{i}+C^{*}\sum_{i=L+1}^{L+U}\xi_{i} \end{array}$$

(6) $$\begin{array}{r} y_{i}\cdot f_{\theta }\left(x_{i}\right)\geq 1-\xi_{i},i=i,\cdots \;,L \end{array}$$

(7) $$\begin{array}{r} |f_{\theta }\left(x_{i}\right)|\geq 1-\xi_{i},i=L+1,\cdots \;,L+U \end{array}$$

(8) $$\begin{array}{r} \xi_{i}\geq 0,i=1,\cdots \;,L+U \end{array}$$

This is a constrained optimization problem if it assumes:

(9) $$\begin{array}{r} \tau \left(y_{i}f_{\theta }\left(x_{i}\right)\right)={\left[1-y_{i}f_{\theta }\left(x_{i}\right)\right]}_{+}=\xi_{i},\xi_{i}\geq 0,i=1,\cdots \;,L \end{array}$$

And

(10) $$\begin{array}{r} \tau^{*}\left(\left|f_{\theta }\left(x_{i}\right)\right|\right)={\left[1-|f_{\theta }\left(x_{i}\right)|\right]}_{+}=\xi_{i},\xi_{i}\geq 0,i=L+1,\cdots \;,\\ L+U \end{array}$$

Then it can be proved that the above-constrained optimization problem is transformed into the following unconstrained optimization problem:

(11) $$\begin{array}{r} {\text{min}}_{w,b}\frac{1}{2}∥\omega^{2}∥+C\sum_{i=1}^{L}\tau \left(y_{i}f_{\theta }\left(x_{i}\right)\right)+C^{*}\sum_{i=L+1}^{L+U}\\ \tau^{*}\left(\left|f_{\theta }\left(x_{i}\right)\right|\right) \end{array}$$

Among them, τ() and τ^*^() are the loss functions of semi-supervised support vector machine (S3VM) with labeled data and unlabeled data, respectively.

In order to speed up the convergence of Stochastic Gradient Descent (SGD), Average Stochastic Gradient Descent (ASGD) performs better and converges faster than ordinary Stochastic Gradient Descent (SGD). Updating the weights is done in the same way as stochastic gradient descent, and can recursively average all previous weights.

(12) $$\begin{array}{r} w_{t+1}=w_{t}-n_{t}\Delta_{w}Q\left(z_{t},w_{t}\right) \end{array}$$

(13) $$\begin{array}{r} {\bar{w}}_{t+1}=\frac{t}{t+1}{\bar{w}}_{t}-\frac{1}{t+1}w_{t} \end{array}$$

When the learning rate η_*t*_ decreases slower than *t*^−1^, ${\bar{w}}_{t}$ converges with the asymptotic rate of optimization. The learning rate η_*t*_ follows formula (14),

(14) $$\begin{array}{r} \eta_{t}=\eta_{0}{\left(1+\lambda \eta_{0}t\right)}^{-0.75} \end{array}$$

η_0_ can be obtained from a small number of experiments on a subset of the training data.

The objective function of this paper is in the form of formula (11). Among them, C and C^*^ are parameters that need to be predefined, and here they are empirically set as

(15) $$\begin{array}{r} C=\frac{1}{L} \end{array}$$

(16) $$\begin{array}{r} C^{*}=\frac{1}{U} \end{array}$$

In this setting, the objective function is differentiated with respect to w to obtain the labeled sample gradient (labeled data):

(17) $$\begin{array}{r} \Delta_{w}Q\left(z_{t},w_{t}\right)=\lambda w_{t}+\frac{dH_{1}}{dw} \end{array}$$

and unlabeled sample gradients (unlabeled data):

(18) $$\begin{array}{r} \Delta_{w}Q\left(z_{t},w_{t}\right)=\lambda w_{t}+\frac{dR_{s}}{dw} \end{array}$$

The weight update formula can be derived as:

(19) $$\begin{array}{r} w_{t+1}=\left(1+\lambda \eta_{t}\right)w_{t}-\eta_{t}\frac{dL}{dw} \end{array}$$

(20) $$\begin{array}{r} {\bar{w}}_{t+1}={\bar{w}}_{t}-\mu_{t}\left(w_{t}-{\bar{w}}_{t}\right) \end{array}$$

Therefore, a semi-supervised support vector machine (S3VM) update round algorithm can be obtained.

## Relationship Between Social Support, Psychological Capital and Mental Health Under Major Epidemics

### Tools and Their Reliability and Validity Tests

This study was conducted using questionnaires. In order to test the discriminant validity of the items, confirmatory factor analysis was used to construct multiple competition models, and the fit indices of the competition models were compared (21, 22). Due to the large number of questions on the scale, the sample size did not reach more than 10 times the number of questions. This study adopted the topic packing strategy. It packs the subscale questions of the two dimensions of “confidence” and “resilience” in the psychological capital scale as two indicators. The subscales of the three dimensions of social support, “objective social support,” “subjective social support” and “utilization of social support” are packaged as three indicators, respectively. The items of the three dimensions of anxiety, depression and stress in the mental health scale are packaged into three indicators. The model fitting results are shown in Table 1. The results showed that the three-factor model fit the best, which verified the discriminant validity of the questionnaire items.

**Table 1:** Discriminant validity and common method bias test results.

**Model**	**CMIN**	**DF**	**CMIN/DF**	**CFI**	**TLI**	**RMSEA**	**SRMR**
Single factor	1252.348	20	62.617	0.591	0.428	0.316	0.229
Two factor	56.996	19	29.842	0.818	0.732	0.216	0.192
Three factor	51.645	17	3.038	0.989	0.981	0.057	0.030
Three factor + method factor	17.708	9	1.968	0.997	0.991	0.04	0.023

To avoid the interference of severe common method bias, the unmeasured potential method factor effect control method was used to test for common method bias. It adds the common method factor to the three-factor model. The fit index of the model did not improve significantly (CFIVTLI did not decrease by more than 0.1, RMSEA and SRMR did not decrease by more than 0.05). This indicates that there is no serious common method bias in this study.

Hypothesis 1: There is a significant positive correlation between psychological capital, social support and mental health under major epidemics;

Hypothesis 2: Psychological capital and social support levels can significantly and positively predict mental health under major epidemics;

Hypothesis 3: Social support plays a partial mediating role between psychological capital and mental health in a major epidemic.

### Psychological Capital, Social Support, Mental Health Status

#### Level of Psychological Capital

Descriptive statistical analysis was performed on the level of psychological capital, and the results are shown in Table 2. The data shows that the average psychological capital is 78.87, which is much larger than the theoretical median. The two sub-dimensions of self-confidence and resilience are 38.71 and 40.16, respectively, with a small difference between the two. The standard deviation of psychological capital score is 13.20, which shows a large degree of dispersion. In general, the psychological capital level of the surveyed group is relatively high, but there are large differences between individuals.

**Table 2:** Descriptive statistics of psychological capital (*N* = 620).

**Variable**	**Minimum**	**Maximum**	**Average**	**Standard deviation**
Psychological capital	16	96	78.87	13.20
Confidence	8	48	38.71	7.28
Toughness	8	48	40.16	6.52

The differences of psychological capital in demographic variables were analyzed, and the results are shown in Table 3.

**Table 3:** Demographic differences analysis of psychological capital.

**Variable**	**Level**	**Average**	**Standard deviation**	**Difference test**
Gender	Male	79.39	13.35	*t* = 0.865
	Female	78.46	13.09	
Grade	One	81.57	10.96	*F* = 43.415
	Two	83.32	11.44	
	Three	81.81	10.94	
	Four	69.82	14.25	
Place of residence	Rural	80.06	12.47	*t* = 4.063
	City	75.05	14.74	
Is it an only child	Yes	79.48	13.23	*t* = 1.004
	No	78.41	13.18	

The results show that there is no significant difference in the level of psychological capital in terms of gender and whether it is an only child. There are significant differences in different grades and places of residence. Among them, the psychological capital level of the fourth grade (M = 69.82) was significantly lower than that of other grades. The psychological capital level of rural students (M = 80.06) was significantly higher than that of urban students. The psychological capital level of students who did not live with their parents (M = 84.47) was significantly higher than that of the other two groups.

#### Analysis of Social Support Status

As shown in Table 4, from the point of view of social support and its various dimension scores, the average score of social support is 45.51, and the standard deviation is 8.44. The mean value of objective support dimension is 10.48, the mean value of subjective support dimension is 26.18, the mean value of social support utilization dimension is 8.84, and the standard deviation is 2.40. On the whole, the social support level obtained by the middle- and lower-grade student groups is higher, the degree of dispersion is lower, and the differences within the group are smaller. From the score of each dimension, objective social support is slightly lower than the theoretical median (11 points). The subjective social support dimension score was much higher than the theoretical median (18 points). The social support utilization dimension score was slightly higher than the theoretical median (6 points).

**Table 4:** Descriptive statistics of social support (*N* = 620).

**Variable**	**Minimum**	**Maximum**	**Average**	**Standard deviation**
Social support	20	68	45.51	8.44
Objective social support	2	24	10.48	3.92
Subjective social support	l1	32	26.18	4.25
Social support utilization	3	12	8.84	2.40

The differences in the demographic variables of social support were analyzed, and the results are shown in Table 5.

**Table 5:** Demographic differences analysis of social support.

**Variable**	**Level**	**Average**	**Standard deviation**	**Difference test**
Gender	Male	44.89	8.73	*t* = −1.628
	Female	46.00	8.19	
Grade	One	46.36	7.81	*F* = 36.072
	Two	49.53	7.92	
	Three	46.43	7.73	
	Four	40.51	7.78	
Place of residence	Rural	45.92	8.58	*t* = 2.184
	City	44.18	7.88	
Is it an only child	Yes	45.62	839	*t* = 0.286
	no	45.42	8.49	

The results showed that there was no significant difference in the level of social support in terms of gender and whether one was an only child. However, there are substantial differences in different grades and places of residence. Among them, the fourth grade's social support level (M = 40.51) is significantly lower than that of other grades. The social support level of students from rural areas (M = 45.92) is significantly higher than that of students from urban areas.

**Figure 9:**
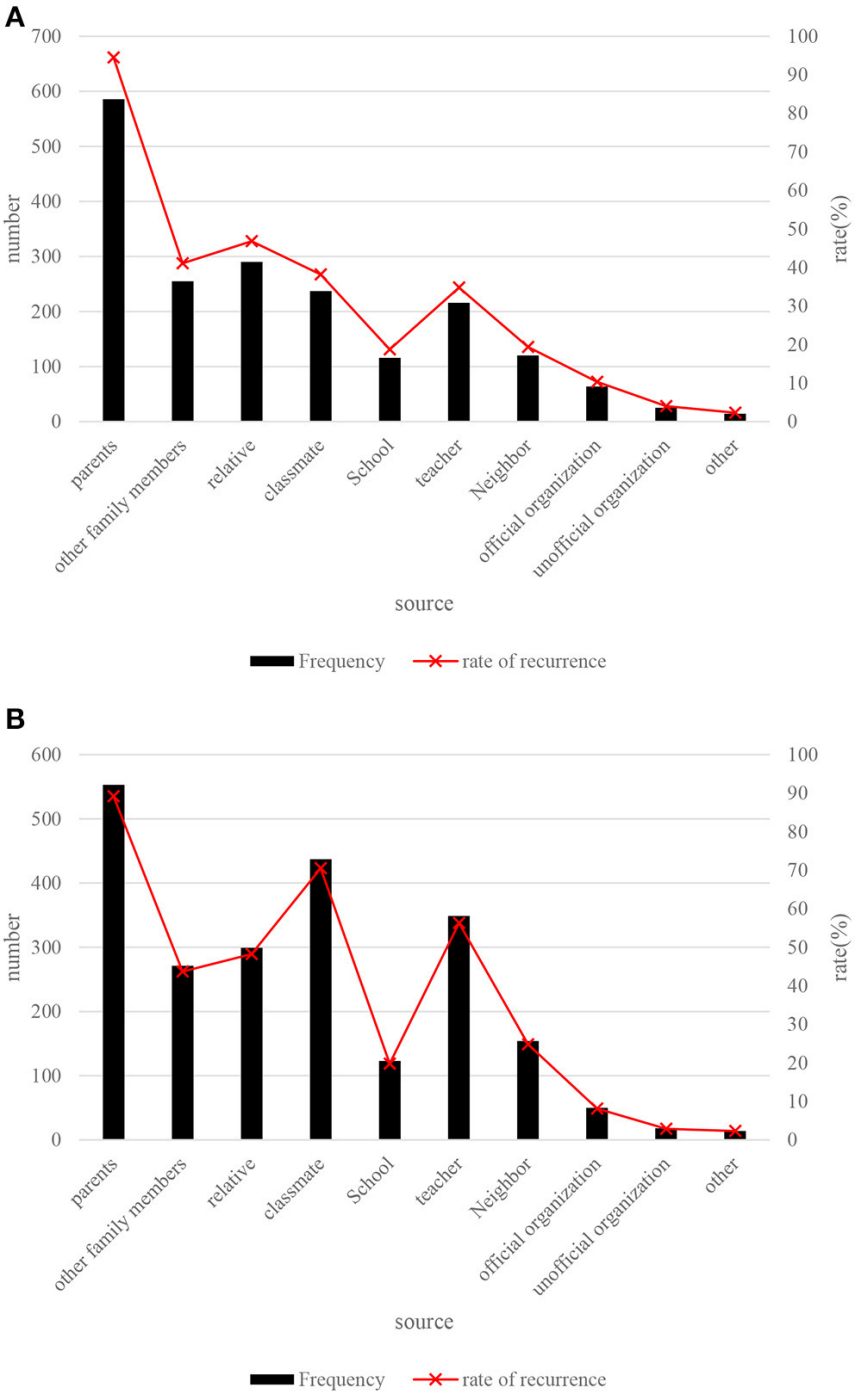
Analysis of the sources of social support. **(A)** Financial support and help in solving practical problems. **(B)** comfort and care.

In order to gain a more detailed understanding of the sources of social support, a frequency analysis was performed for each option of items 7 and 8. Items 7 and 8 select sources for “financial support and help with practical problems” and “comfort and concern.” The specific analysis results are shown in Figure 9.

The results in Figure 9 show that the most important sources of social support for college students are parents, relatives and other family members, followed by classmates and teachers, followed by neighbors and schools. From the perspective of the types of social support, “economic support and help in solving practical problems” mainly belong to material support, and “comfort and care” belong to emotional support. Therefore, the material support and emotional support obtained from parents, relatives and family members are more, and the emotional support obtained from classmates and teachers is more than material support.

#### Analysis of Mental Health Status

In order to understand the mental health status of college students under major epidemics, descriptive statistical analysis was first conducted on the total score of mental health and the three dimensions of anxiety, depression and stress symptoms. The results are shown in Table 6.

**Table 6:** Descriptive statistics of mental health (*N* = 620).

**Variable**	**Minimum**	**Maximum**	**Average**	**Standard deviation**
Mental health	21	84	32.32	13.66
Anxiety	7	28	10.99	4.57
Depression	7	28	11.41	5.09
Stress	7	28	9.92	4.82

It can be seen from the analysis results that the average mental health score is 32. 32, slightly higher than the theoretical median (31. 5), with a standard deviation of 13.66. It shows that college students have certain mental health problems in the major epidemic situation. From the score of each dimension, the score of anxiety and depression dimension is higher than that of stress dimension, which reflects that the level of anxiety and depressive symptoms is more common in college students than stress symptoms.

On this basis, the differences in the psychological health level of demographic variables were analyzed, and the analysis results are shown in Table 7.

**Table 7:** Demographic differences analysis of mental health.

**Variable**	**Level**	**Average**	**Standard deviation**	**Difference test**
Gender	Male	32.60	14.11	*t* = 0.449
	Female	32.10	13.30	
Grade	One	30.22	12.76	*F* = 3.812
	Two	3.30	14.30	
	Three	32.36	14.22	
	Four	35.09	13.02	
Place of residence	Rural	31.97	13.85	*t* = −1.128
	City	33.43	13.00	
Is it an only child	Yes	31.89	13.33	*t* = −0.686
	No	32.65	13.91	

The results showed that there were no significant differences in the level of mental health in terms of gender, place of residence, and whether they were an only child. There are significant differences in different grades and living conditions with parents. Among them, the mental health symptom score of seniors (M = 40.51) was significantly higher than that of freshman and sophomore students, and the score was also higher than that of juniors, but it was not significant.

### Mediating Model of Psychological Capital, Social Support and Mental Health

Commonly used test methods for mediating effects include four-step method, Sobel test and Bootstrap confidence interval method. However, the four-step method has low test power for the mediating effect. The Sobel test is susceptible to sample size and distribution normality. The principle of the Bootstrap method is to perform repeated sampling from an original sample and replace it again. The size of the sample drawn is the same as the original sample. The values of N statistics are obtained from N samples, and the Bootstrap distribution of the statistics is formed, avoiding the influence of non-normal distribution. The test of the mediation effect is mainly aimed at the statistic ab, and the confidence interval of ab is obtained (usually set to 95%). If the interval does not contain 0, the mediation effect in the model is significant.

As shown in Table 8, the results of the mediation effect test show that the direct path from psychological capital to mental health is not significant. The mediating path of psychological capital to mental health through social support was significant (*p* < 0.001). It shows that social support plays a complete mediating role, and the effect size of the mediation model reaches 49.70%.

**Table 8:** Test of the mediating effect of social support on mental health.

**Effect**	**Effect size**	**95% confidence interval**		**The proportion of mediation effect**	**Amount of effect**
		**Upper limit**	**Lower limit**		
Direct effect	−0.051	0.128	−0.240	79.50%	49.70%
Mediation effect	−0. 194	−0.068	−0.333		
Total effect	−0.244	−0.113	−0.364		

**Figure 10:**
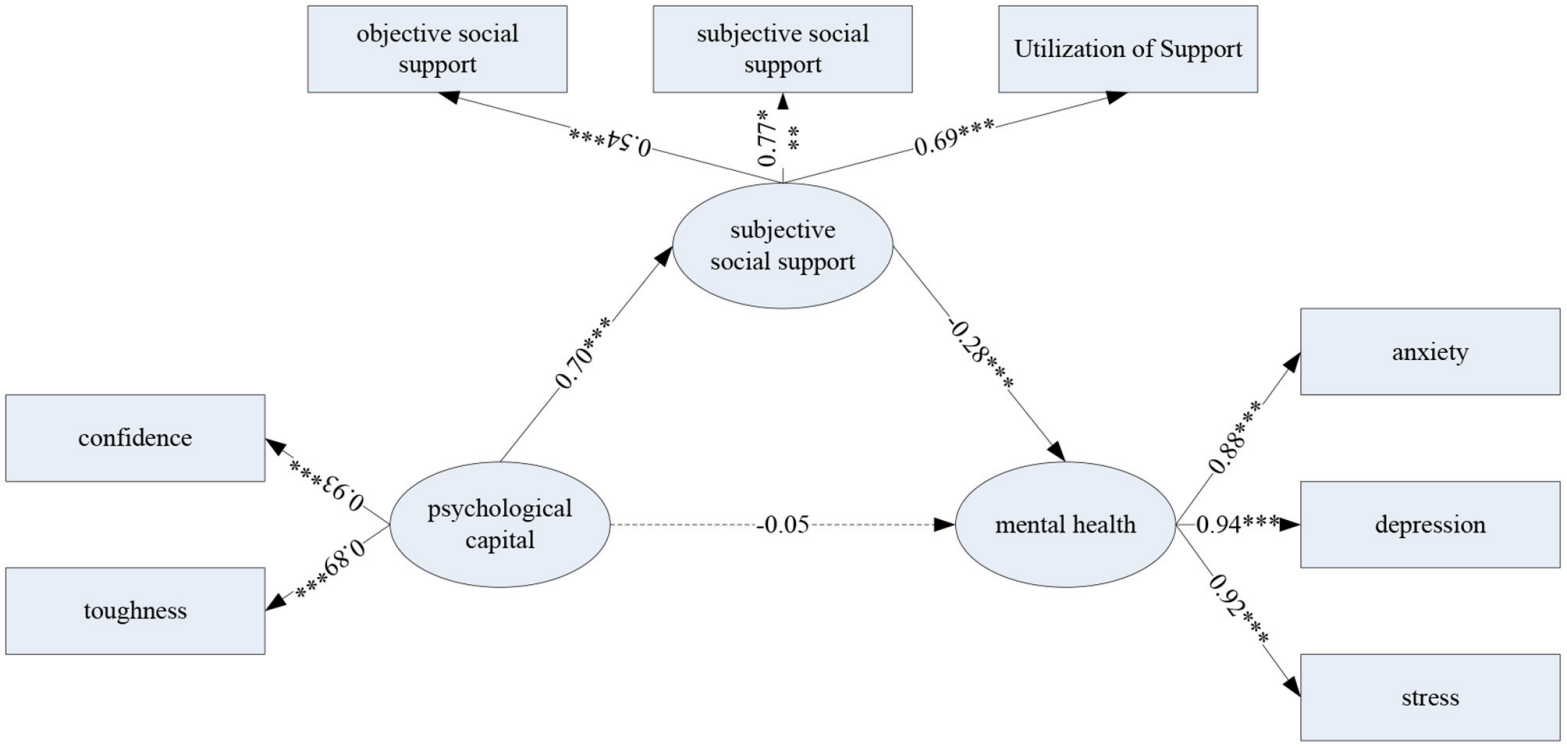
The mediating effect model of psychological capital and social support on mental health.

The specific path coefficients of the mediation model are shown in Figure 10.

In terms of mental health status, the overall mental health problems of the surveyed groups are not serious, but they cannot be ignored. In particular, the analysis of differences in demographic variables shows that, from the perspective of different learning stages of students, freshmen, sophomores, juniors and seniors have significant differences in mental health. Seniors have the worst mental health. The reason for such a result is mainly because the lower grade students do not have the pressure of employment and further education. However, senior students are faced with the enormous pressure of employment and postgraduate entrance examination, as well as the uncertainty of future development after graduation, which lead to their mental health facing many challenges in practice. Coupled with the epidemic's impact, their depression, anxiety and stress conditions are not optimistic. Especially if the opportunity is not seized during the employment period, some graduates may lose the opportunity of employment. For them, this pressure can be imagined. Of course, in the lower grades, the learning tasks of the junior and senior students are relatively heavy. Therefore, their psychological pressure and other three dimensions are significantly worse than the freshman and second graders. In addition, other factors during the pandemic can negatively impact the mental health of college students. As the epidemic lasted for a very long time, students spent a long time at home. In order to prevent the epidemic, many parents have restricted students from going out during the epidemic. As a result, students have very limited space for activities in family life, and they can only pass the time by watching TV, playing with mobile phones, etc. It is difficult to gain a sense of value and achievement from various activities, which will negatively affect their positive and healthy psychological state.

Psychological teachers and counselors can increase the chances of successfully obtaining social support by helping college students cultivate a higher level of psychological capital and enhance their tendency and ability to use social support. At the same time, families, schools, and society should objectively provide sufficient material or emotional, social support so that social support can have a greater role in promoting mental health.

## Conclusions

From the perspective of developmental psychology, college students are still in the stage of psychological development and are gradually participating in social integration. Compared with adults who have formed mature social, behavioral abilities and established a relatively stable social network, college students are still in a dynamic socialization process. In this process, changes in social support will have a more obvious impact on college students. For example, there is an attachment relationship with parents during childhood, gradually transitioning to adolescence, adapting to the social resources provided by teachers, classmates, institutions, etc., and forming new social relationships. Good social resource acquisition and social communication process help them to form a more positive cognition of interpersonal relationships and social environment, gain a higher sense of trust and security, etc., thus promoting the development of mental health. At the same time, good teacher-student relationships and family function are also positive factors for college students to obtain and feel social support, which is helpful for students' social adaptation. In the long run, this will contribute to the mental health development of college students.

## Data Availability Statement

The original contributions presented in the study are included in the article/supplementary material, further inquiries can be directed to the corresponding author.

## Author Contributions

HY was conceived of the study and participated in its design, as well as supervised the study, and critically revised the manuscript. XL wrote the manuscript. Both authors read and approved the final version of the manuscript.

## Funding

This work was supported by Anhui Provincial Education Quality Engineering Project under grant no. 2020jyxm0143 and Anhui Polytechnic University Research Project under grant no. Xjky2022207.

## Conflict of Interest

The authors declare that the research was conducted in the absence of any commercial or financial relationships that could be construed as a potential conflict of interest.

## Publisher's Note

All claims expressed in this article are solely those of the authors and do not necessarily represent those of their affiliated organizations, or those of the publisher, the editors and the reviewers. Any product that may be evaluated in this article, or claim that may be made by its manufacturer, is not guaranteed or endorsed by the publisher.
